# Supplementary material for: Rate and oscillatory switching dynamics of a multilayer visual microcircuit model
Source: eLife. 2022 Aug 22;11:e77594. doi: 10.7554/eLife.77594 (PMC9395191; doi:10.7554/eLife.77594)
Supplement: Supplementary file 1. — (a) Connection probabilities for different morphologically defined cell types, as described in Jiang et al., 2015. BC: basket cells; ChC: chandelier cells; MC: Martinotti cells; BTC: bitufted cells; SC: shrub cells; HEC: horizontally elongated cells; BPC: bipolar cells. (b) EPSP/IPSP strength for different morphologically defined cell types, as described in Jiang et al., 2015. BC: basket cells; ChC: chandelier cells; MC: Martinotti cells; BTC: bitufted cells; SC: shrub cells; HEC: horizontally elongated cells; BPC: bipolar cells. (c) Morphological interneuron types, their genetic marker and proportion. (d) Connectivity matrix corrected for cell proportions and scaled up by G = 100. (e) Time constants for different neuron types. [file elife-77594-supp1.docx]

**Supplementary Table 1 (Supplementary file 1a)**

|  | | | presynaptic | | | | | | | | | | |
| --- | --- | --- | --- | --- | --- | --- | --- | --- | --- | --- | --- | --- | --- |
|  |  |  | L 2/3 | | | | | L 5 | | | | | |
|  |  |  | **PYR** | **BC** | **ChC** | **MC** | **BTC** | **PYR** | **BC** | **SC** | **HEC** | **MC** | **BPC** |
| Postsynaptic | L2/3 | **PYR** | 0.02 | 0.35 | 0.31 | 0.44 | 0.18 | --- | 0.1 | --- | --- | 0.21 | --- |
|  |  | **BC** | 0.19 | 0.47 | --- | 0.49 | 0.03 | 0.03 | 0.22 | --- | --- | 0.25 | --- |
|  |  | **ChC** | 0.19 | 0.18 | --- | --- | 0.26 | --- | 0.03 | --- | --- | --- | --- |
|  |  | **MC** | 0.2 | 0.11 | --- | 0.48 | 0.14 | --- | 0.03 | --- | --- | 0.3 | --- |
|  |  | **BTC** | --- | 0.14 | 0.38 | 0.46 | --- | --- | --- | --- | --- | 0.27 | --- |
|  | L5 | **PYR** | 0.04 | 0.06 | --- | 0.08 | --- | 0.02 | 0.25 | 0.1 | 0.3 | 0.21 | --- |
|  |  | **BC** | 0.08 | 0.14 | --- | 0.05 | --- | 0.11 | 0.48 | 0.03 | --- | 0.35 | --- |
|  |  | **SC** | 0.11 | 0 | --- | 0 | --- | 0.08 | 0.16 | --- | --- | --- | 0.31 |
|  |  | **HEC** | --- | 0.04 | --- | 0.44 | --- | --- | --- | --- | --- | 0.31 | --- |
|  |  | **MC** | --- | 0.02 | --- | --- | --- | --- | 0.13 | 0.63 | --- | 0.34 | --- |
|  |  | **BPC** | 0.08 | 0.04 | --- | --- | --- | 0.17 | 0.04 | --- | 0.5 | 0.33 | --- |

**Supplementary Table 2 (Supplementary file 1b)**

|  | | | presynaptic | | | | | | | | | | |
| --- | --- | --- | --- | --- | --- | --- | --- | --- | --- | --- | --- | --- | --- |
|  |  |  | L 2/3 | | | | | L 5 | | | | | |
|  |  |  | **PYR** | **BC** | **ChC** | **MC** | **BTC** | **PYR** | **BC** | **SC** | **HEC** | **MC** | **BPC** |
| postsynaptic | L2/3 | **PYR** | 0.3 | -0.5 | -0.35 | --- | -0.3 | --- | --- | -0.4 | --- | --- | -0.25 |
|  |  | **BC** | 1.6 | -0.7 | --- | --- | -0.2 | --- | 1 | -0.5 | --- | --- | -0.4 |
|  |  | **ChC** | 0.9 | -0.9 | --- | --- | -0.3 | --- | --- | -0.2 | --- | --- | 0 |
|  |  | **MC** | 1.3 | -0.4 | --- | --- | -0.4 | --- | --- | -0.3 | --- | --- | -0.7 |
|  |  | **BTC** | 0 | -0.69 | -0.47 | --- | --- | --- | --- | --- | --- | --- | -0.54 |
|  | L5 | **PYR** | 1.15 | -0.56 | --- | --- | --- | --- | 0.53 | -0.43 | --- | -0.81 | -0.45 |
|  |  | **BC** | 1.1 | -0.2 | --- | --- | --- | --- | 0.3 | -0.8 | -0.44 | -0.9 | -0.3 |
|  |  | **SC** | 1.3 | -0.8 | --- | --- | --- | --- | 1.2 | -1.2 | -0.53 | --- | -0.4 |
|  |  | **HEC** | 0.5 | --- | --- | -0.3 | --- | -0.3 | 0.5 | -0.4 | --- | --- | 0 |
|  |  | **MC** | --- | -0.4 | --- | --- | --- | --- | --- | --- | --- | --- | -0.4 |
|  |  | **BPC** | --- | -0.25 | --- | --- | --- | --- | --- | -0.6 | -1.4 | --- | -0.48 |

**Supplementary Table 3 (Supplementary file 1c)**

| Name | Genetic Marker | Proportion |
| --- | --- | --- |
| Basket Cell | PV | L2/3: 40% L5: 32% |
| Chandelier Cell | PV | L2/3: 2% |
| Shrub Cell | PV | L5: 18% |
| Horizontally-Elongated Cell | PV | L5: 10% |
| Martinotti Cell | SST | L2/3: 11% L5:32% |
| Bipolar Cell | VIP | L2/3: 17% |
| Bitufted Cell | VIP | L2/3: 10% |

**Supplementary Table 4 (Supplementary file 1d)**

|  | *presynaptic* | | | | | | | | | |
| --- | --- | --- | --- | --- | --- | --- | --- | --- | --- | --- |
| *postsynaptic* |  | | *L 2/3* | | | | *L5* | | | |
|  |  |  | **PYR** | **PV** | **SST** | **VIP** | **PYR** | **PV** | **SST** | **VIP** |
|  | *L2/3* | **PYR** | 0.6 | -1.44 | -0.29 | -0.11 | --- | -0.26 | -0.34 | --- |
|  |  | **PV** | 30.4 | -2.7 | -0.54 | -0.01 | 3 | -0.7 | -0.64 | --- |
|  |  | **SST** | 17.1 | -1.3 | --- | -0.16 | --- | -0.04 | --- | --- |
|  |  | **VIP** | 26 | -0.35 | -0.53 | -0.11 | --- | -0.06 | -1.34 | --- |
|  | *L5* | **PYR** | 4.4 | -0.1 | -0.04 | --- | 0.6 | -1.98 | -0.4 | --- |
|  |  | **PV** | 10.4 | -0.9 | -0.02 | --- | 13.2 | -7.73 | -0.9 | --- |
|  |  | **SST** | 5.5 | --- | --- | --- | 4 | -0.41 | --- | -0.32 |
|  |  | **VIP** | --- | -0.13 | -0.58 | --- | --- | --- | -0.79 | --- |

**Supplementary Table 5 (Supplementary file 1e)**

| Time Constants | Value |
| --- | --- |
| $\boldsymbol{\tau}_{\mathbf{PYR}}$ | 3 ms |
| $\boldsymbol{\tau}_{\mathbf{PV}}$ | 7 ms |
| $\boldsymbol{\tau}_{\mathbf{SST}}$ | 30 ms |
| $\boldsymbol{\tau}_{\mathbf{VIP}}$ | 10 ms |
