# Supplementary material for: Rate and oscillatory switching dynamics of a multilayer visual microcircuit model
Source: eLife. 2022 Aug 22;11:e77594. doi: 10.7554/eLife.77594 (PMC9395191; doi:10.7554/eLife.77594)
Supplement: Source code 1. [file elife-77594-code1.zip › 03-02-2022-RA-eLife-77594/Source_code_1.docx]

**Matlab model code written in version R2020b**

clear all

% This code provides ascending and descending input to VIP cells and

% plots the response of all cell types in superficial (sup) and deep

% layers (inf), as shown in Fig. 3

% connectivity matrix

C = [0.006, -0.0144, -0.0029, -0.001, 0, -0.0026, -0.0034, 0;

0.304, -0.027, -0.00539, -0.00012, 0.03, -0.007, -0.0064, 0;

0.171, -0.01296, 0, -0.0016, 0, -0.0004, 0, 0;

0.26, -0.0035, -0.0053, -0.0011, 0, -0.0006, -0.01344, 0;

0.044, -0.00096, -0.0004, 0, 0.006, -0.01978, -0.004, 0;

0.104, -0.0089, -0.0002, 0, 0.132, -0.0773, -0.00896, 0;

0.055, 0, 0, 0, 0.04, -0.0041, 0, -0.0032;

0, -0.0013, -0.0058, 0, 0, 0, -0.0079, 0];

%g-value

g = [150];

%simulation duration in seconds

duration = 2;

%noise level

sigma = 0;

%synaptic time constants

tauE_sup = 0.003;

tauPV_sup = 0.007;

tauSST_sup = 0.03;

tauVIP_sup = 0.01;

tauE_inf = 0.003;

tauPV_inf = 0.007;

tauSST_inf = 0.03;

tauVIP_inf = 0.01;

% baseline external input

IextE_sup_baseline = 5;

IextPV_sup_baseline = 0;

IextSST_sup_baseline = 5;

IextVIP_sup_baseline = 0;

IextE_deep_baseline = 5;

IextPV_deep_baseline = 0;

IextSST_deep_baseline = 5;

IextVIP_deep_baseline = 0;

N_scale_vector_up = 1:1:100;

N_scale_vector_down = 100:-1:1;

columns = 1;

units = 8;

dt = 0.0001;

ds = 1;

tau_vector = [tauE_sup; tauPV_sup; tauSST_sup; tauVIP_sup; tauE_inf; tauPV_inf; tauSST_inf; tauVIP_inf];

r(1:units,1) = 0.01;

c_index = 0;

C_scaled = C.* g;

index = 0;

Rate_vector_up = zeros(units,numel(N_scale_vector_up));

Rate_vector_down = zeros(units,numel(N_scale_vector_down));

for n_up = N_scale_vector_up

% external input

IextE_sup = IextE_sup_baseline ;

IextPV_sup = IextPV_sup_baseline;

IextSST_sup = IextSST_sup_baseline;

IextVIP_sup = IextVIP_sup_baseline + n_up;

IextE_inf = IextE_deep_baseline;

IextPV_inf = IextE_deep_baseline;

IextSST_inf = IextE_deep_baseline;

IextVIP_inf = IextE_deep_baseline + n_up;

Iext_vector = [IextE_sup; IextPV_sup; IextSST_sup; IextVIP_sup; IextE_inf; IextPV_inf; IextSST_inf; IextVIP_inf];

[Rate, Time] = microcircuit_model(duration, columns, units, C_scaled, tau_vector, Iext_vector, sigma, dt, ds, r);

index = index + 1;

Rate_vector_up(1:units,index) = Rate(1:units,10000);

r = Rate(:,size(Rate,2));

end

index = 0;

for n_down = N_scale_vector_down

% external input

IextE_sup = IextE_sup_baseline;

IextPV_sup = IextPV_sup_baseline;

IextSST_sup = IextSST_sup_baseline;

IextVIP_sup = IextVIP_sup_baseline + n_down;

IextE_inf = IextE_deep_baseline;

IextPV_inf = IextE_deep_baseline;

IextSST_inf = IextE_deep_baseline;

IextVIP_inf = IextE_deep_baseline + n_down;

Iext_vector = [IextE_sup; IextPV_sup; IextSST_sup; IextVIP_sup; IextE_inf; IextPV_inf; IextSST_inf; IextVIP_inf];

[Rate, Time] = microcircuit_model(duration, columns, units, C_scaled, tau_vector, Iext_vector, sigma, dt, ds, r);

index = index + 1;

Rate_vector_down(1:units,index) = Rate(1:units,10000);

r = Rate(:,size(Rate,2));

end

figure;

colors = ['b', 'r', 'g', 'm', 'b', 'r', 'g', 'm'];

title_plot = ["PYR-sup" "PV-sup" "SST-sup" "VIP-sup" "PYR-deep" "PV-deep" "SST-deep" "VIP-deep"];

plot_index = 0;

for x = 1:8

plot_index = plot_index + 1;

subplot(2,4,plot_index)

plot(N_scale_vector_down, Rate_vector_down(x,:), 'k')

hold on

plot(N_scale_vector_up, Rate_vector_up(x,:), colors(x))

title(title_plot(x))

if or(plot_index == 1, plot_index==5)

ylabel('rate')

end

if plot_index>=5

xlabel('Iext to VIP')

end

end
