# Supplementary material for: Rate and oscillatory switching dynamics of a multilayer visual microcircuit model
Source: eLife. 2022 Aug 22;11:e77594. doi: 10.7554/eLife.77594 (PMC9395191; doi:10.7554/eLife.77594)
Supplement: Source code 2. [file elife-77594-code2.zip › 03-02-2022-RA-eLife-77594/Source_code_2.docx]

function [rate, time] = microcircuit_model(duration, columns, units, C, tau_vector, Iext_vector, sigma, dt, ds, r)

%time steps;

t_steps = 0:dt:duration;

D = length(t_steps);

%tau_vector

tau_all = repmat(tau_vector,columns,1);

%external_input_vector

Iext_all = repmat(Iext_vector, columns,1);

%noise_vector

sigma_all = repmat(sigma, columns*units,1);

% transfer functions:

F = @(x) x./(1 - exp(-x/1));

%downsampling

D_ds = length(0:ds*dt:duration)-1;

time = 0:ds*dt:(D_ds-1)*ds*dt;

rate = zeros(columns*units,D_ds);

tt = 0;

%Model simulation

for t = 1:D

u = C*r + Iext_all;

K = feval(F,u);

r = r + dt*(-r + K)./tau_all + sqrt(dt)*sigma_all.*randn(columns*units,1);

r = abs(r);

if mod(t,ds)==0

tt=tt+1;

rate(:,tt) = r;

end

end
